# Pharmacists’ Attitudes and Practices Regarding Tetanus, Diphtheria and Pertussis (Tdap) Vaccination in Pregnancy and Surrounding Newborns

**DOI:** 10.3390/pharmacy6020036

**Published:** 2018-04-25

**Authors:** Christine A Echtenkamp, Stacie J Lampkin, Amany K Hassan

**Affiliations:** D’Youville School of Pharmacy, D’Youville School, Buffalo, NY 14201, USA; Christine_Echtenkamp@URMC.Rochester.edu (C.A.E.); hassana@dyc.edu (A.K.H.)

**Keywords:** Diphtheria-Tetanus-acellular Pertussis Vaccines, vaccination, pregnancy, infant, newborn

## Abstract

Background: Bordetella pertussis or whooping cough is a serious and vaccine-preventable illness. Despite widespread vaccination in the pediatric population, pertussis still infects approximately 100,000 infants each year in the United States. The purpose of this study was to determine gaps in pharmacists’ understanding, attitudes, practices, and barriers surrounding the tetanus, diphtheria, and pertussis (Tdap) vaccination recommendation for patients who are pregnant or planning to come in close contact with infants. Methods: This study was a descriptive, exploratory electronic survey. The survey assessed three major areas; the role of the pharmacist in Tdap vaccination, perceived barriers to vaccination, and understanding the recommendations. Results: A total of 225 pharmacists responded to the survey. Pharmacists who responded to this survey agreed that pharmacists should have a role vaccinating the public and individuals expecting to come into contact with a newborn, (88.5% and 86.9%) respectively, but fewer agreed that pharmacists should have a role vaccinating pregnant women against tetanus, diphtheria, and pertussis (77%, *p* < 0.001). Based on the responses to case scenarios, only 22.5% and 30.6% of respondents understood the recommendations. Numerous barriers to vaccinating pregnant women were identified. Conclusion: While most pharmacists surveyed felt they should have a role in vaccinating pregnant women and those expecting to come in contact with a newborn, there are barriers to implementing this practice. Future efforts should focus on further evaluating identified gaps and developing programs for pharmacists that emphasize the significance of vaccinating these patients to reduce the burden of pertussis in infants.

## 1. Introduction

Bordetella pertussis or whooping cough is a serious and vaccine-preventable illness that poses a significant morbidity and mortality risk to children under the age of two [1]. Despite widespread vaccination in the pediatric population, pertussis still infects approximately 100,000 infants each year in the United States [2]. The updated 2013 recommendation by the Advisory Committee on Immunization Practices (ACIP) states that women should receive the tetanus, diphtheria, acellular pertussis (Tdap) vaccination in their third trimester between 27 and 36 weeks gestation with each pregnancy [3,4]. It is also recommended that all people who are expected to come in contact with an infant receive one dose of the Tdap vaccine if not previously vaccinated, as cocooning provides maximal protection to the infant. This should ideally be administered at least two weeks prior to contact [3].

According to the American College of Obstetricians and Gynecologists (ACOG), in order to confer protection to infants who are at risk for pertussis infection (i.e., have not yet received or are not able to receive their primary vaccine series), it is essential pregnant women and close contacts of infants receive the Tdap vaccine as recommended by the ACIP [4]. Recommendation by a trusted health care professional has been shown to be one of, if not the most important factor, influencing a woman’s decision to receive vaccinations while pregnant [5]. In addition, a 2012 study found that 45% versus 83% of adults not vaccinated who have come in, or are expecting to come in, contact with an infant would consider getting the Tdap vaccine if recommended by a family member versus recommended by a healthcare profession. Thus, as experts in medication information and safety, pharmacists are well positioned to provide information and influence the vaccination related decisions of pregnant women and close contacts of infants [6].

In recent years, state laws and policies have shifted to allow pharmacists to administer more vaccines, which have been shown to improve immunization rates [7]. As the pharmacists’ scope of vaccination practice expands, it is essential for pharmacists to follow and make recommendations based on the most current vaccination guidelines, including making recommendations for patients who are pregnant. To the best of our knowledge, there are no studies evaluating pharmacists’ understanding, attitudes, and practices of the Tdap vaccination recommendation during pregnancy and for close contacts of newborns as set forth by the ACIP. This descriptive, non-experimental study assessing pharmacists in this area will assist in identifying gaps and lead to development of strategies to address gaps and barriers surrounding vaccination of pregnant females and close contacts of infants.

The objective of this study was to evaluate pharmacists’ understanding, attitudes, and practices surrounding the ACIP Tdap vaccination recommendation for patients who are pregnant or planning to come in close contact with infants. In addition, we sought to identify barriers to implementing these recommendations in patients who are pregnant.

## 2. Materials and Methods

The survey for this study was distributed electronically via Qualtrics^®^ on 12 April 2016 to a random sample of 10,000 licensed pharmacists across the United States. Of surveys sent, 317 were undeliverable, and 9683 pharmacists successfully received the survey via email. Surveys were sent to pharmacists who practiced within community, mass merchandise, chain, and independent pharmacies according to the SK&A database. An email was sent inviting pharmacists to participate in the study within two weeks of receipt by completing the survey accessed on the web by way of a provided hyperlink. A reminder email was sent on 20 April 2016 to all individuals who had not yet completed the survey, and the survey closed on 26 April 2016.

The survey questions were modeled after a previously published study evaluating pharmacists’ knowledge, attitudes, and practices regarding influenza vaccination in pregnant women, and modified to apply to Tdap vaccination practices [5]. The survey had 27 questions that assessed three major areas; the role of the pharmacist in vaccinating against tetanus, diphtheria, and pertussis, perceived barriers to vaccination, and understanding of the ACIP recommendations. The survey questions consisted of multiple-choice questions, open-ended questions, statements which required a response on a 3-point Likert-type scale (agree, neutral, disagree), and case-based questions that asked participants to select all choices that applied. The case-based questions tested respondents knowledge of recommendations by requiring participants to respond to case scenarios pertaining to use of the Tdap vaccine in pregnant females or close contacts of infants. In addition, the survey collected baseline respondent characteristics including: gender, state of practice, year of graduation, practice environment, and completion of immunization certification. The original survey was tested for face validation by authors of the previous study using convenience sampling to test wording and format, and to identify areas needing clarification [5]. The modified survey was tested again among pharmacists in Buffalo, NY selected via convenience sampling, to check for grammatical and clerical errors and ensure the comprehensibility of the questions prior to the survey deployment.

The survey took, on average, ten minutes to complete. Survey participation was voluntary and anonymous, and no personal identifiers were collected. As an incentive to complete the survey, participants were given the opportunity to provide their name and email address in order to be entered in a drawing for a $100 VISA gift card. The D’Youville Institutional Review Board approved the study.

The number of U.S. pharmacists in 2015 was 282,000, thus we estimated that 400 respondents were needed based on a 5% margin of error and 95% confidence interval [8]. Assuming a 6.8% response rate and accounting for invalid email addresses, we targeted 10,000 pharmacists via email [9]. Unfortunately, the actual response rate to our survey was less than reported in previous studies [5]. Since trends were found in the data and further funding was unable to be obtained to get resources to continue this survey, the study was stopped with the current response rate.

Data were analyzed using SPSS version 23.0 (IBM Corp. Released 2015. IBM SPSS Statistics for Windows, Version 23.0. Armonk, NY, USA: IBM Corp.). Demographic characteristics of respondents were summarized using frequencies and percentages. Chi-square tests were used to test differences in responses among participants by gender, year of graduation, and role in the pharmacy. McNemar’s tests were used to measure the level of agreement in responses to outcomes of interest. Geographical regions were categorized as: South, Midwest, Northeast, and West. Primary job functions were categorized as community (staff pharmacist and manager/supervisor), clinical (clinical pharmacist and resident/post graduate), and other (retired, director, assistant director, and consultant pharmacist). Year of graduation was categorized as pharmacists graduating before the year 2000 (prior to the Doctor of Pharmacy degree being the required standard) and during or after the year 2000 (year the Doctor of Pharmacy degree became required). A *p*-value of <0.05 was considered statistically significant. Values and percentages were calculated based on the number of respondents for each question, which varied between 173 and 225 throughout the survey.

## 3. Results

There were 9,683 valid emails sent successfully. A total of 225 pharmacists responded to the survey and 173 pharmacists completed the survey accounting for the varying number of respondents to individual questions within the results of the survey. The response rate for the survey was 2.3%. Demographics are reported in Table 1.

### 3.1. Attitudes of Vaccinating in Pregnancy

One hundred sixty-two (88.5%) respondents agreed that pharmacists have an important role to play in vaccinating the public against tetanus, diphtheria, and pertussis. Of those respondents, only 140 (76.5%) also agreed that pharmacists have an important role in vaccinating pregnant patients against tetanus, diphtheria, and pertussis (*p* < 0.001). Overall 141 (77.1%) agreed, 30 (16.4%) were neutral, and 12 (6.6%) respondents disagreed that “pharmacists have an important role to play in vaccinating pregnant females against tetanus, diphtheria, and pertussis”. The majority of respondents agreed that vaccinating women during pregnancy will protect the newborn against tetanus, diphtheria, and pertussis; 153 (89.5%). Additionally, 148 (81%) respondents agreed that pharmacists should offer the Tdap vaccination to pregnant women if legally authorized to do so. However, only 126 (68.9%) of those pharmacists also agreed that the vaccine is safe to administer to patients who are pregnant. Overall, when asked if, “the Tdap vaccination is safe during pregnancy,” 148 (80.9%) respondents agreed and 35 (19.1%) either disagreed or were neutral.

Comparing those who graduated prior to the year 2000 with those who graduated during or after the year 2000, no statistically significant difference was found on agreement with the statements that pharmacists have a role to play in vaccinating the public (85.7% vs. 98.2% agreed) and pregnant females (71.4% vs. 84.9% agreed) against tetanus, diphtheria, and pertussis. There were no significant differences in opinion on the safety of the vaccine in pregnancy among respondents graduating prior to or after the year 2000 (77.6% vs. 83.6%). Specific to immunization training, 91 (82%) pharmacists who had completed a training course and 42 (68.9%) pharmacists who had not completed a training course agreed that pharmacists should have a role vaccinating pregnant females (*p* = 0.049). There were no significant differences between these two groups with regard to having a role in vaccinating the public (91.9% vs. 83.6% agreed) or the safety of the vaccine in pregnancy (83.8% vs. 73.8% agreed). Table 2 provides a comparison by type of pharmacist.

### 3.2. Practices and Barriers to Vaccinating in Pregnancy

One hundred thirty-two (72.1%) participants responded that they routinely ask women who are pregnant if they have received or plan to receive the Tdap vaccine during their pregnancy. Ninety-four (51.4%) respondents reported that they vaccinate patients against tetanus, diphtheria, and pertussis; however, only 71 (38.8%) reported they vaccinate pregnant patients with Tdap (*p* < 0.001). Summarized in Table 3 are the top reasons for vaccinating pregnant patients with Tdap as reported by pharmacists who currently vaccinate these women. The barriers to vaccinating pregnant women against tetanus, diphtheria, and pertussis selected by respondents are reported in Table 4. Respondents were also given the opportunity to comment via free text response. Several respondents expressed concerns regarding the safety of the vaccination, commenting that a lack of communication between pharmacists and obstetricians may result in duplicate vaccination and stressing the need for involvement of obstetricians in the vaccination of these patients.

### 3.3. Understanding of Recommendations During Pregnancy

To evaluate pharmacists understanding of the 2013 ACIP recommendations, respondents were presented with three case scenarios and asked which action(s) they would take. The results can be found in Table 5. Additionally, among the 86 (49.7%) pharmacists who stated they would make a recommendation to the 21-week pregnant female, 29 (33.7%) of those individuals would also offer the vaccination that day.

### 3.4. Attitudes, Practices, and Understanding Recommendations in Close Contact to Newborns

One hundred fifty-nine (86.9%) respondents agreed that pharmacists have an important role to play in providing Tdap vaccination to those who are expected to come in contact with newborns. When comparing responses by graduation year, 81 (82.7%) respondents graduating prior to the year 2000, and 68 (93.2%) graduating during or after 2000 agreed that pharmacists have a role to play in vaccinating those who are expecting to come in contact with a newborn infant against tetanus, diphtheria, and pertussis (*p*-value = 0.043). When comparing responses among pharmacists who had completed an immunization-training course versus those who had not, 102 (91.9%) and 48 (78.7%), respectively, agreed that pharmacists should have a role vaccinating patients expecting to come in contact with a newborn (*p* = 0.013). To assess understanding of the ACIP recommendation, a case-based scenario was presented in which a 59 year old male comes into the pharmacy with a prescription for atorvastatin and tells you that he is looking forward to visiting with his newborn granddaughter. One hundred thirty-two (76.3%) pharmacists stated that they would ask the patient if his Tdap vaccinations were up to date and 87 (50.3%) said that they would offer the vaccine today.

## 4. Discussion

Since the 2013 Tdap vaccination recommendations were established, Tdap vaccination rates in pregnant women and close contacts of infants have not been reported. However, a 2015 Center for Disease Control (CDC) report examining vaccination rates in 2011 across 15 states found that only 55.7% of women with live births reported receiving a Tdap vaccine [10] demonstrating the need to increase vaccination rates in pregnant females. Evidence demonstrates pharmacists have a significant role in vaccinating the public and increasing other vaccination rates [7]. Our study identified trends in knowledge gaps among pharmacists and barriers to immunization that need to be further evaluated and addressed in order to maximize the utility of pharmacists and improve vaccination rates in this patient population.

Most pharmacists who responded to our survey agreed that pharmacists should play a role in vaccinating the public (88.5%) and those expecting to come in contact with a newborn against tetanus, diphtheria, and pertussis. Significantly fewer respondents agreed that pharmacists should have a role in the vaccination of pregnant women (77.1%) against tetanus, diphtheria, and pertussis. The results of a study evaluating pharmacists’ knowledge and attitudes regarding influenza vaccination found that most pharmacists who completed an immunization training program agreed that they should have a role in vaccinating the public and pregnant patients against influenza (97% and 82%) [5]. Similarly, our study found that pharmacists who completed an immunization training program were likely to agree that pharmacists should have a role in vaccinating the public and pregnant patients against tetanus, diphtheria, and pertussis (92% and 82%). Our study further evaluated the attitudes and practices of pharmacists who had not completed an immunization training program. While no significant differences were found in their opinion on the role of pharmacists in immunizing the public against tetanus, diphtheria, and pertussis, fewer respondents agreed that pharmacists should have a role in vaccinating pregnant patients with the Tdap vaccination. The significant variability between respondents who felt that pharmacists should have a role in vaccinating pregnant patients against tetanus, diphtheria, and pertussis (77%), and those who actually ask pregnant patients if they have or plan to receive a Tdap vaccine (72%) and who are vaccinating pregnant patients (39%) further supports the need to address the identified barriers to vaccinating pregnant patients.

Pharmacists in our study identified numerous barriers to implementing Tdap vaccination recommendations in pregnant women. The most significant barriers recognized, in order of significance, were a lack of insurance coverage, pregnant women are not interested in being vaccinated, doctors are not recommending the vaccination, and liability concerns. Lack of insurance coverage for pharmacists vaccinating patients is not a new barrier and not specific to patients who are pregnant. While coverage may vary widely between pregnant females and can impact their access to the vaccination, this topic was beyond the scope of our study and requires further investigation. It is notable that increasing health care insurance coverage in the U.S. has historically been identified as a means to increasing vaccination rates among other vaccines [11].

The second most significant barrier identified in our study was pregnant women not being interested in vaccination. Previous studies have also established that there are a variety of factors that affect the attitudes of pregnant women towards vaccination, including varying degrees of perceived severity of the diseases, safety concerns, limited time, cost concerns, and opinions of friends and health care professionals [5,12]. These perceptions have been identified as opportunities for pharmacists to overcome barriers and serve a health care need.

Our survey did not identify safety as one of the top five barriers, even though similar studies seeking to identify barriers to vaccinating pregnant women across the health care system and examining various immunizations have consistently identified safety concerns as a major barrier. When specifically asked about safety, 19% of pharmacists had concerns about the safety of Tdap during pregnancy. Concerns about safety increased in pharmacists who reported their primary practice area as clinical pharmacy and for those who did not complete a training course. Data available fails to identify any significant safety risks associated with the vaccine. Neither the Vaccine Adverse Events Reporting System (VAERS) or adverse event reporting systems established by Tdap vaccination manufacturers have received any reports of adverse events [13]. As of 2015, Food and Drug Administration (FDA) pregnancy categories have been removed by the pregnancy and lactation labeling rule in an effort to better assist health care professionals in weighing the risks versus benefits of medication and vaccination use in pregnant patients. However, since the Tdap vaccination was approved prior to the labeling rule, the manufacturer-specific FDA pregnancy category B/C drug is still available as a reference in vaccine information resources [14,15]. Due to these concerns about the vaccine, future efforts to increase awareness about the safety and efficacy among the community and health care professionals is warranted. Respondents also expressed concerns regarding the safety of the vaccination, commenting that a lack of communication between pharmacists and obstetricians may result in duplicate vaccination and stressing the need for coordination with an obstetrician. Future efforts could be geared towards forming and assessing collaboration agreements between pharmacists and obstetricians.

Even if the aforementioned barriers are overcome, ensuring that the ACIP recommendations are followed is an important concern. Responses to the case-based questions suggest additional training for pharmacists may be necessary to improve pharmacists’ understanding of the current ACIP recommendations. When asked about a case scenario involving a woman 21 weeks pregnant, who should not receive the vaccination for at least 5 weeks per the ACIP recommendations, 50% said that they would make a recommendation regarding the Tdap vaccination in pregnancy. Of those respondents who said they would recommend Tdap vaccination to this woman, nearly one-third stated that they would offer the vaccine today, suggesting they would have incorrectly recommended the woman be vaccinated at 21 weeks pregnant. Furthermore, when asked about patients 29 and 31 weeks pregnant, within the recommended vaccination window, little difference was seen in the number of respondents who would offer the vaccination at that time versus the 21 week pregnant patient. The case scenario involving a 29-week pregnant female also identified her as having another 14-month-old child for whom she was picking up an antibiotic. Though the patient may have presumably been vaccinated during her previous pregnancy, this should not influence the recommendation, as a Tdap vaccination should be received with every pregnancy. These findings demonstrate the potential to increase awareness of the recommendation, and this topic should be emphasized in vaccination training programs, and recommendations in pregnancy should be highlighted in immunization schedules.

The majority of respondents agreed that pharmacists should have a role in vaccinating patients who expect to come in contact with a newborn against tetanus, diphtheria, and pertussis. Most pharmacists who responded to the study agreed that pharmacists should have a role in vaccinating those who come in contact with newborns against tetanus, diphtheria, and pertussis (86%) and 76% of respondents said they would ask the patient if his Tdap vaccinations were up to date. A study published in 2014 demonstrated the ability of pharmacist-administered immunizations and vaccine advocacy efforts to increase vaccine coverage per live births from 5.5% to 8.1% [16]. This may serve as an ideal opportunity for pharmacists to improve immunization rates.

The low response rate of our study limited the ability to generalize results to all pharmacists, but it had similar demographics (age, gender, work schedule) to a study examining pharmacist’s attitudes and practices regarding influenza vaccination in pregnant women [5]. However, we received more responses from clinical pharmacists (23% of respondents) who may have different knowledge of the recommendations compared to community pharmacists. Despite differences in primary job function and the relatively low response rate, our study was able to identify barriers and trends amongst pharmacist understanding of and attitudes toward the 2013 ACIP recommendation; thus, providing a foundation to stem future research. Another limitation of our study was that it was unable to account for variability in state laws, although regardless of state-specific legislation, all pharmacists in the United States are legally able to provide recommendations and education regarding vaccination in pregnancy.

## 5. Conclusions

Pharmacists have an important role to play in vaccinating pregnant women and those expecting to come in contact with a newborn against tetanus, diphtheria, and pertussis. While our survey findings suggest that most pharmacists agree that they should have a role in vaccinating these populations, it also found a need to increase awareness and overcome barriers to pharmacists immunizing these patients. Despite ACIP recommendations, the health care community consistently identifies a need to improve immunization rates among pregnant females. To best equip pharmacists to meet this health care need, future efforts should focus on further evaluating identified gaps and developing programs emphasizing the significance of vaccinating these patients to reduce the burden of pertussis in infants.

## Figures and Tables

**Table 1:** Respondent pharmacist characteristics (*N* = 225).

Characteristic	*n* (%)
Gender	
Male	66 (38)
Female	105 (61)
Other	1 (1)
Work schedule	
Full time	147 (85)
Part time	25 (15)
Graduation year	
<2000	98 (57)
≥2000	73 (43)
Pharmacy location *	
Northeast	47 (21)
Midwest	79 (35)
South	60 (26)
West	41 (18)
Practice area	
Rural	39 (23)
Suburban	73 (42)
Urban	60 (35)
Primary job function	
Director/Assistant director	17 (10)
Clinical Pharmacist	39 (23)
Consultant Pharmacist	1 (<1)
Manager/Supervisor	44 (26)
Post graduate/Resident	1 (<1)
Retired	1 (<1)
Staff Pharmacist	69 (40)

* Northeast: CT, MA, ME, NH, NJ, NY, PA, RI, VT; Midwest: IA, IL, IN, KS, MI, MN, MO, ND, NE, OH, SD, WI; South: AL, AR, DC, DE, PL, GA, KY, LA, MD, MS, NC, OK, SC, TN, TX, VA, WV; West: AK, AZ, CA, CO, HI, ID, MT, NM, NV, OR, UT, WA, WY.

**Table 2:** Agreement on role perceptions of pharmacists by area of practice.

Primary Area of Practice	Clinical Pharmacy	Community Pharmacy	Other	*p*-Value
Pharmacists should have a role vaccinating the public against Tdap *n* (%)	32 (82.1)	105 (92.9)	25 (80.6)	0.059
Pharmacists should have a role vaccinating pregnancy females against Tdap *n* (%)	25 (64.1)	98 (86.7)	18 (58.1)	<0.001
The Tdap vaccination is safe during pregnancy *n* (%)	26 (66.7)	100 (88.5)	22 (71)	0.094
Pharmacists should have a role vaccinating those who expect to come into contact with a newborn against Tdap *n* (%)	31 (79.5)	103 (91.2)	25 (80.6)	0.004

**Table 3:** Reasons respondents immunize pregnant women with Tdap.

Reason to Immunize	*n* (%)
*N*	69
It is the right thing to do	60 (87)
It complies with CDC guidelines	50 (72)
It expands my scope of practice	44 (64)
It expands my skill set	40 (58)
It provides professional development for pharmacists	35 (51)
It increases business	31 (45)
I like the individual patient contact	30 (43)
I am required to by my employer	27 (39)
It strengthens relationships with local physicians	24 (35)
It helps recruit pharmacists to work in our store	5 (7)

**Table 4:** Reported barriers to vaccinating pregnant women against Tetanus, Diphtheria, and Pertussis.

Barrier	*n* (%)
*N*	173
Patients insurance will not cover it	70 (41)
Pregnant women are not interested in being vaccinated	45 (26)
Doctors are not advising pregnant women to be vaccinated	35 (20)
Liability	35 (20)
My pharmacy’s corporate policy/protocol does not allow it	25 (14)
I do not feel confident in recommending the vaccine	19 (11)
Insufficient safety data	19 (11)
Pregnant women don’t want to wait in line	14 (8)
Insufficient compensation	11 (6)
Physician should be responsible for vaccinating	4 (2)
Vaccine is not available in general	3 (2)
Supply of the vaccine is limited	2 (1)
I do not feel there are any barriers	30 (17)

**Table 5:** Evaluating pharmacists’ understanding of the recommendations.

Case Scenario Summary *N* = 183	21 Week Pregnant Female; Comes to Pharmacy to Pick Up Prenatal Vitamins	29 Week Pregnant Female; Comes to Pharmacy to Pick Up Antibiotic for 14 Month Old Son	31 Week Pregnant Female; Comes to Pharmacy to Pick Up Prenatal Vitamins
Ask patient if they received a Tdap vaccine during this pregnancy *n* (%)	70 (40.5)	75 (43.4)	81 (46.8)
Ask patient if they are aware of the recommendations for Tdap vaccination in pregnancy *n* (%)	100 (57.8)	69 (39.9)	85 (49.1)
Make a recommendation regarding Tdap vaccination in pregnancy *n* (%)	86 (49.7)	69 (39.9)	76 (43.9)
Offer the Tdap vaccination today *n* (%)	30 (17.3)	39 (22.5)	53 (30.6)
